# Lung Function in Fontan Patients Over a Ten-Year Period: Is the Fontan Circulation Impairing Lung Development?

**DOI:** 10.1007/s00246-023-03389-2

**Published:** 2024-01-25

**Authors:** Maren Ravndal, Lars Idorn, Kim Gjerum Nielsen, Vibeke Hjortdal

**Affiliations:** 1 Department of Cardiothoracic Surgery, Copenhagen University Hospital Rigshospitalet, Copenhagen, Denmark; 2 Section of Pediatric Cardiology, Department of Pediatrics and Adolescent Medicine, Rigshospitalet, Copenhagen University Hospital, Copenhagen, Denmark; 3 Pediatric Pulmonary Service, Department of Pediatrics and Adolescent Medicine, Copenhagen University Hospital, Copenhagen, Denmark; 4 Department of Clinical Medicine, University of Copenhagen, Copenhagen, Denmark

**Keywords:** Congenital heart disease, Fontan circulation, Lung function, Pulmonary function test, Lung development, Cohort study

## Abstract

Few studies have investigated how the Fontan circulation affects lung function, and no studies have investigated the development of lung function over longer time in these patients. We aimed to describe the development of lung function in Fontan patients over a 10-year period. Pulmonary function tests (PFT), including spirometry and diffusion capacity for Carbon Monoxide (DLCO) and Nitric Oxide (DLNO), were conducted in a Danish Fontan cohort in 2011 (PFT-I). In 2021, re-investigations were performed (PFT-II). We investigated changes in percent predicted (%pred) lung function from PFT-I to PFT-II. Patients were categorized into a pediatric group (age under 18 at PFT-I) and an adult group (age 18 or older at PFT-I). Out of the 81 patients completing PFT-I, 48 completed PFT-II. In the pediatric group (32 patients), there were significant declines in %pred forced expiratory volume in 1s (99.7 (92.4, 104.4)–89.3 (84.9, 97.2), *p* < 0,001), forced vital capacity (98.3 (87.8, 106.1)–96.7 (86.7, 100.6), *p* = 0.008), and alveolar volume (95.5 (89.5, 101.6)–89.5 (79.7, 93.2), *p* < 0.001). The corresponding measurements remained stable in the adult group. However, the median %pred DLNO significantly declined in the adult group (58.4 (53.3, 63.5)–53.7 (44.1, 57.3), *p* = 0.005). Over a 10-year period, several lung function parameters declined significantly in the younger Fontan patients, suggesting possible impairments in lung development during growth. The decline in %pred DLNO in the adult patient group indicates deterioration of the membrane component of diffusion capacity, implying that the Fontan circulation might negatively affect the alveolar membrane over time.

**Supplementary Information:**

The online version contains supplementary material available at 10.1007/s00246-023-03389-2.

## Introduction

Since the introduction of the Fontan procedure in 1971, improvements in surgical and medical treatment have dramatically increased the long-term survival for patients with univentricular hearts. Consequently, most patients will now survive beyond early adulthood [1]. However, most will also experience complications [2]. Fontan-associated complications can affect multiple organs. Some complications are well described, such as arrhythmias, thromboembolic events, Fontan-associated liver disease, and impaired exercise capacity [1]. The lungs are another critical organ susceptible to complications in Fontan circulation. This circulatory system can lead to various pulmonary flow disturbances, which may vary depending on the specific heart defect and the location along the surgical pathway. For all patients, a series of open-heart surgeries in childhood ultimately result in a circulation where the systemic venous return is directly connected to the pulmonary circulation, giving a non-pulsatile, decreased pulmonary blood flow. Given the immense development of the lungs during childhood and adolescence [3, 4], it is plausible that the establishment of a Fontan circulation with its major changes in pulmonary blood flow during the first years of life can affect long-term lung function [5–7].

The number of studies investigating lung function in Fontan patients is limited, with a majority of older reports [8–14]. Existing studies acknowledge impairments in lung function among Fontan patients, but as far as we know, no previous study has described the development in lung function over time. There is also a lack of updated data with use of more recent reference equations on the diffusion capacity and its components in Fontan patients.

The primary objective of this study was to conduct a comprehensive investigation of lung function in Fontan patients by (a) describing the development in lung function over a 10-year period; (b) describing the diffusion capacity for Carbon Monoxide (DLCO) and Nitric Oxide (DLNO) using the most updated reference equations; and (c) investigate the relationship between lung function parameters and clinical parameters, including VO2_peak_. We hypothesized that the lung function would decline with increasing age in the Fontan patients, primarily with a decline in the pulmonary diffusion capacity, secondary to the abnormal hemodynamics of the Fontan circulation.

## Material and Methods

### Patients

A Danish national Fontan study was conducted in 2011, including pulmonary function tests (PFT)—hereby referred to as PFT-I. All eligible Fontan patients followed at Copenhagen University Hospital, Rigshospitalet, were invited to PFTs, which included measurements of lung volumes, DLCO, and DLNO. A new Danish national Fontan study was conducted in 2021, where the same patients were re-invited to a new PFT, hereby referred to as PFT-II. Patients were recruited through the outpatient clinic at Copenhagen University Hospital, Rigshospitalet. Only patients who completed all PFT measurements in both PFT-I and PFT-II were included in the final analyses. Updated medical history was systematically retrieved from the patient’s medical records, and echocardiography findings from the latest examination were also obtained. As part of routine clinical control for all patients, the ejection fraction was visually assessed by an experienced investigator and reported as normal or mildly, moderately, or severely reduced. As part of the national Fontan studies in both 2011 and 2021, patients were also invited to a cardiopulmonary exercise test (CPET). CPET results are published in a separate manuscript [15], but selected data from the CPETs in 2021 are also presented here—to identify possible associations between PFT measurements and VO2_peak_. The study was approved by the National Scientific Ethics Committee (H-20028226), and all patients provided written informed consent before the investigations.

### Pulmonary Function Tests

Methods for PFT-I are previously described [14]. In short, PFT-I was performed in a sitting position with use of the Jaeger Masterscreen PFT Pro. In PFT-II, spirometry, DLCO, and DLNO measurements were again conducted using the Jaeger Masterscreen PFT pro (CareFusion, Hoechberg, Germany). The patients utilized a disposable mouthpiece with an integrated bacterial/viral filter (Spirobach, Tyco, Healthcare, Italy), which was connected to the pneumotachograph. The procedures for spirometry and DLCO were conducted in accordance with the latest ATS/ERS guidelines [16–18]. For DLCO, a 10-s breath-hold was applied, and we considered a ratio between inspiratory volume and FVC (VIN/FVC) above 80% as sufficient, deviating from the conventional threshold of 85%. This adjustment is in keeping with the method in a previous publication assessing DLCO and DLNO [19]. Regarding DLNO, the procedure followed the latest recommendations, with the use of the same technique as in DLCO [18, 20], but with a breath-hold time of 5 s. The DLCO was measured both in the 10-s procedure (DLCO_10s_), and in the 5-s procedure (DLCO_5s_). The following measurements were obtained: forced expiratory volume in 1 s (FEV1), forced vital capacity (FVC), FEV1/FVC ratio, alveolar volume (VA), DLCO, DLCO/VA (DLCO corrected for alveolar volume), DLNO, DLNO/VA (DLNO corrected for alveolar volume), and DLNO/DLCO ratio. Furthermore, the simultaneous single-breath measurement of DLCO and DLNO allows for calculation of the membrane diffusing capacity (Dm) and the capillary blood volume (Vc) [21]. The Dm and Vc measurements of the DLNO are of interest since they make up the two subcomponents of diffusing capacity of the lungs, and by differentiating them, one can estimate whether a reduced diffusing capacity is primarily driven by damage to the alveolar capillary membrane, or by reductions in capillary blood volume [20, 22, 23]. When interpreting our results, we adhered to the lates assumptions of CO’s and NO’s reaction rates with hemoglobin [20]. Because DLNO has a much faster reaction with Hemoglobin (Hb) compared to DLCO, DLNO is primarily dependent on the integrity of the alveolar membrane (measured as Dm), while DLCO is primarily dependent on the available amount of Hb in the lung capillaries (measured as Vc) [24].

### The Global Lung Initiative Calculator for Spirometry, Lung Volume, and DLCO

The Global Lung Function Initiative (GLI) online calculator for spirometry [25], DLCO [26], and lung volumes [27] was applied for the absolute measurements from both PFT-I and PFT-II. The GLI calculator is based on a large material of reference data from around the world. The GLI calculator was not available at PFT-I, why the percent predicted (%pred) values at that time were calculated with older equations, which today are considered outdated. It was, therefore, reasonable to interpret the data from PFT-I again, using the GLI calculator. While the GLI calculator provides valid and continuous %pred values for spirometry, lung volumes, and DLCO for ages 3–95 years, we chose to categorize our patients into two separate groups: a pediatric group comprising patients under 18 years at PFT-I and an adult group comprising patients 18 years or older at PFT-I. This decision was made to categorize patients into the younger patients where we expected a further lung development in the study period, in contrast to the adult patients, where lung development likely was close to complete at PFT-I. Also, a cut-off at 18 years was chosen to allow for use of adult reference equations on DLNO results from both PFT-I and PFT-II in the adult group.

### DLNO Reference Equations

DLNO results from PFT-I were also calculated and interpreted again, using more recent reference equations. For patients 18 years or over, calculations of %pred values for DLNO, DLNO/VA, Dm, and Vc in both PFT-I and PFT-II were made with the reference equations provided by Zavorsky et al. [20]. For patients under 18 years, the reference equations provided by Thomas et al. [19] were used. However, these two sets of reference equations are designed for either adult or pediatric patients. When we compared the %pred DLNO results in patients transitioning from childhood to adulthood during the study period, there were significant discrepancies in the %pred values at the shift from the pediatric to the adult equations. To ensure consistency, with use of the same reference equation for DLNO results from both PFT-I and PFT-II, we only included the adult group (18 years or older at PFT-I) in the final longitudinal DLNO analyses.

### Comparison with a Danish Pediatric Reference Material

The categorization of a pediatric group also allowed for comparison of cross-sectional data from PFT-I between the pediatric Fontan patients and published pediatric reference data from Thomas et al. [19]. The reference material comprises results from spirometry, lung volumes, DLCO, and DLNO in 297 healthy Danish children. These data were not available at PFT-I, and a new interpretation of the DLNO results in the pediatric Fontan group was considered well reasoned. While longitudinal analyses of the DLNO results in the pediatric group were not feasible, a comparison of the absolute DLNO data from PFT-I between the pediatric Fontan group and the healthy Danish children allowed new insight into the DLNO results.

### Statistical Analysis

Comparisons of baseline clinical characteristics between the pediatric group and the adult group were performed using a *t* test for continuous variables, or a Fisher exact test for categorical variables. Comparison of PFT-data and clinical characteristics in PFT-I and PFT-II were analyzed using appropriate statistical tests based on the data distribution; paired *t* test or Wilcoxon Signed Rank test was employed for continuous data, while McNemar’s test was used for categorical data. The results were reported as means ± standard deviation (SD) or medians and interquartile range (IQR), depending on the distribution. Univariate regression models were conducted to explore the association between lung parameters and clinical characteristics. For significant findings, a subsequent multivariate analysis was performed to account for potential confounding factors, including age, sex, and alveolar volume. To compare the impact of age on PFT measurements between the pediatric Fontan patients and the Danish pediatric reference material, we conducted an interaction analysis using regression. Statistical significance was defined as a *p*-value < 0.05 for all tests conducted. All statistical analyses were performed using RStudio version 2022.07.

## Results

### Patients

In PFT-I, there were 103 eligible Fontan patients followed at Copenhagen University Hospital, after the exclusion of patients under 6 years. Out of these, 87 patients were recruited, with completion of spirometry, DLCO, and DLNO in 81 patients. In PFT-II, 6 of the 81 patients were not eligible, and 27 were not included because of various reasons, please see Flowchart 1. This left 48 patients (18 females) for final inclusion—with complete, valid measurements in both PFT-I and PFT-II. When categorizing the patients into the pediatric and the adult groups, there were 32 patients in the pediatric group (age under 18 years at PFT-I) and 16 patients in the adult group (age 18 or older at PFT-I). The mean time interval between the two PFTs was 10.9 ± 0.9 years. In the pediatric group, the median ages at PFT-I and PFT-II were 12 and 23 years, respectively, while in the adult group, the corresponding median ages were 22 and 32 years. The baseline characteristics for the two groups are presented in Table 1. Except for a higher median age at Fontan completion in the adult group (8 years vs. 2 years, *p* < 0.001), there were no significant differences in baseline characteristics between the two groups. For both the pediatric and the adult group, lateral tunnel was the most common Fontan type, while tricuspid atresia and double inlet left ventricle were the most common diagnoses. Left ventricular morphology was present in 63% of the patients in both groups. In Table 2, the changes in clinical characteristics from PFT-1 and PFT-II are presented, with *p* values indicating any significant changes in each characteristic within each group. The only significant change in complications was an increase in the number of pediatric patients with atrioventricular valve regurgitation from PFT-I to PFT-II.

**Flowchart 1:**
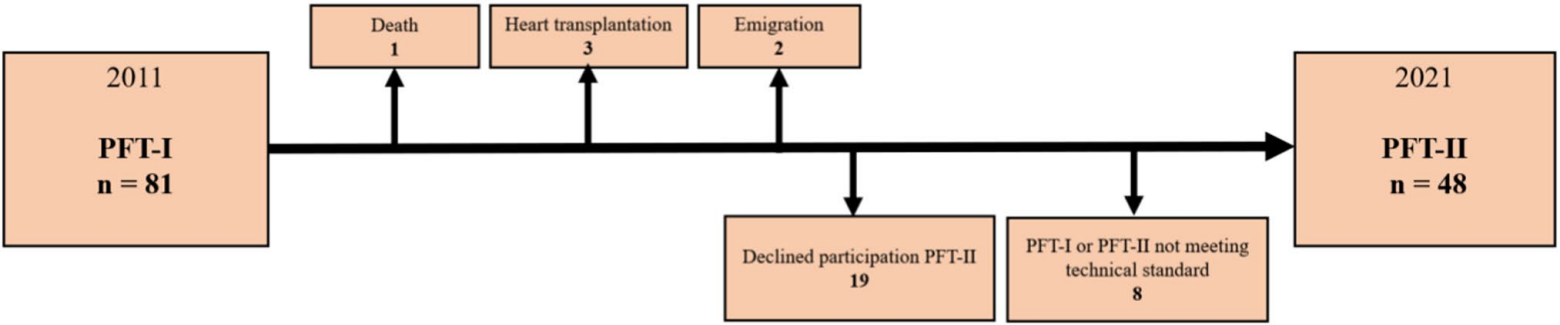
Flowchart presenting patient inclusion in PFT-I and PFT-II

**Table 1:** Patient characteristics

	*n* (%)		*p*
	Pediatric group (*n* = 32)	Adult group (*n* = 16)	
*Sex*			0.545
Females	11 (34.4)	7 (43.8)	
Males	21 (65.6)	9 (56.2)	
*Systemic ventricle*			1.000
Left ventricular morphology	20 (62.5)	10 (62.5)	
Right ventricular morphology	12 (37.5)	6 (37.5)	
*Fontan type*			0.192
Extracardiac conduit	11 (34.4)	4 (25)	
Lateral tunnel	21 (65.6)	10 (62.5)	
Classical Fontan	0	2 (12.5)	
*Age at Fontan completion*			**<0.001**
Years, median (IQR)	2 (1, 3)	8 (5, 10)	
*Diagnoses*			0.937
Tricuspid atresia	9 (28.1)	3 (18.8)	
Double inlet left ventricle	7 (21.9)	4 (25.0)	
Unbalanced atrioventricular valve defect	4 (12.5)	3 (18.8)	
Hypoplastic left heart syndrome	5 (15.6)	2 (12.5)	
Other	7 (21.9)	4 (25.0)	

Significance levels less than 0.05 are presented as bold

Patients are categorized into a pediatric group (under 18 years at PFT-I) and an adult group (18 years or older at PFT-I)

*PFT* pulmonary function test

**Table 2:** Clinical characteristics at PFT-I and PFT-II

	Pediatric group (*n* = 32)			Adult group (*n* = 16)		
	PFT-I	PFT-II	*p*	PFT-I	PFT-II	*p*
*Age* (years), median (IQR)	12 (9, 15)	23 (17, 26)		22 (20, 26)	32 (30, 37)	
*Height* (cm), mean ± SD	148 ± 16.7	176 ± 7.9		172.4 ± 7.9	176.5 ± 6.9	
*BMI*, mean ± SD	18.33 ± 3.4	23.9 ± 4.4		22.7 ± 3.9	26.1 ± 5.4	
*Ejection fraction, echocardiography*			0.617^a^			1.000^a^
Normal	22 (68.8)	20 (62.5)		8 (50)	7 (43.8)	
Mildly reduced	10 (31.3)	9 (28.1)		5 (31.3)	6 (37.5)	
Moderately reduced	0 (0)	3 (9.4)		2 (12.5)	2 (12.5)	
Severely reduced	0 (0)	0 (0)		1 (0.7)	1 (0.7)	
*Atrioventricular valve regurgitation, echocardiography*			**0.003**^**b**^			0.480^b^
None	15 (46.9)	4 (12.5)		4 (25.0)	2 (12.5)	
Mild	14 (43.8)	26 (81.3)		10 (62.5)	12 (75.0)	
Moderate	4 (6.3)	2 (6.3)		2 (12.5)	2 (12.5)	
Severe	1 (3.1)	0 (0)		0 (0)	0 (0)	
*Arrhythmia*			0.248			1.000
	10 (31.3)	13 (40.6)		7 (43.8)	8 (0.5)	
*Pacemaker*						
	4 (12.5)	7 (21.9)	0.248	3 (18.8)	4 (0.25)	1.000

Significance levels less than 0.05 are presented as bold

Patients are categorized into a pediatric group (under 18 years at PFT-I) and an adult group (18 years or older at PFT-I)

Data expressed as *n* (%), unless otherwise specified

*PFT* pulmonary function test, *BMI* body mass index

^a^Normal ejection fraction versus mildly/moderately/severely reduced ejection fraction

^b^No atrioventricular valve regurgitation versus mild/moderate/severe atrioventricular valve regurgitation

### Pulmonary Function Tests in the Pediatric Group

**Fig. 1:**
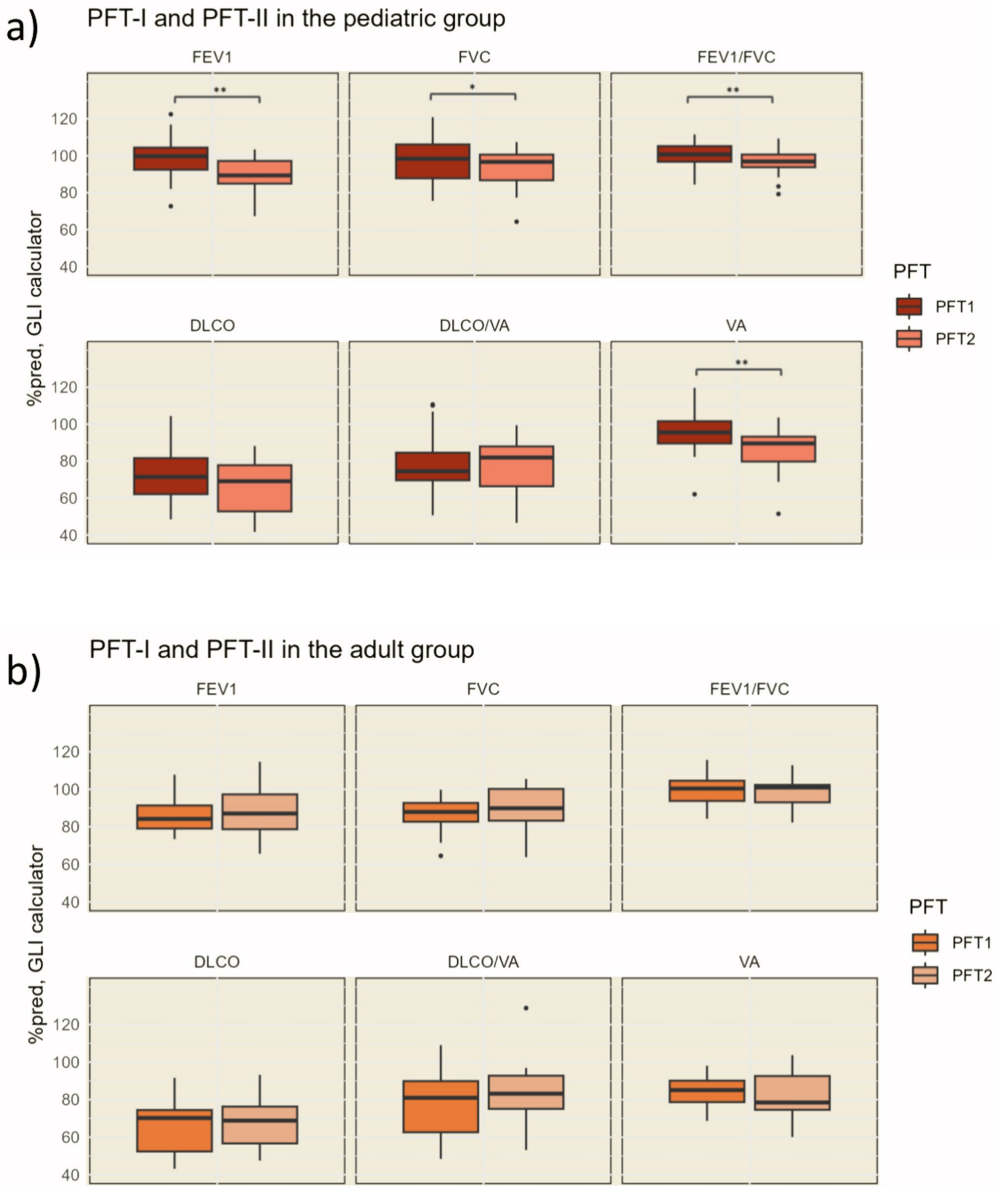
Results from spirometry, lung volume, and DLCO_10s_ in PFT-I and PFT-II, presented as percent predicted (%pred). Patients are categorized into a pediatric group (under 18 years at PFT-I), results presented in, and an adult group (18 years or older at PFT-I). All %pred values are calculated with the GLI calculator. Wilcoxon signed-rank test for paired data was conducted to check for significant differences from PFT-I to PFT-II within each group (**p < 0.001,*p < 0.05). **a** Results from PFT-I and PFT-II in the pediatric group. **b** Results from PFT-I and PFT-II in the adult group. *FEV1* forced expiratory volume in 1 s, *FVC* forced vital capacity, *DLCO* diffusing capacity for carbon monoxide, *DLCO/VA* coefficient of the lung for carbon monoxide, *VA* alveolar volume

**Fig. 2:**
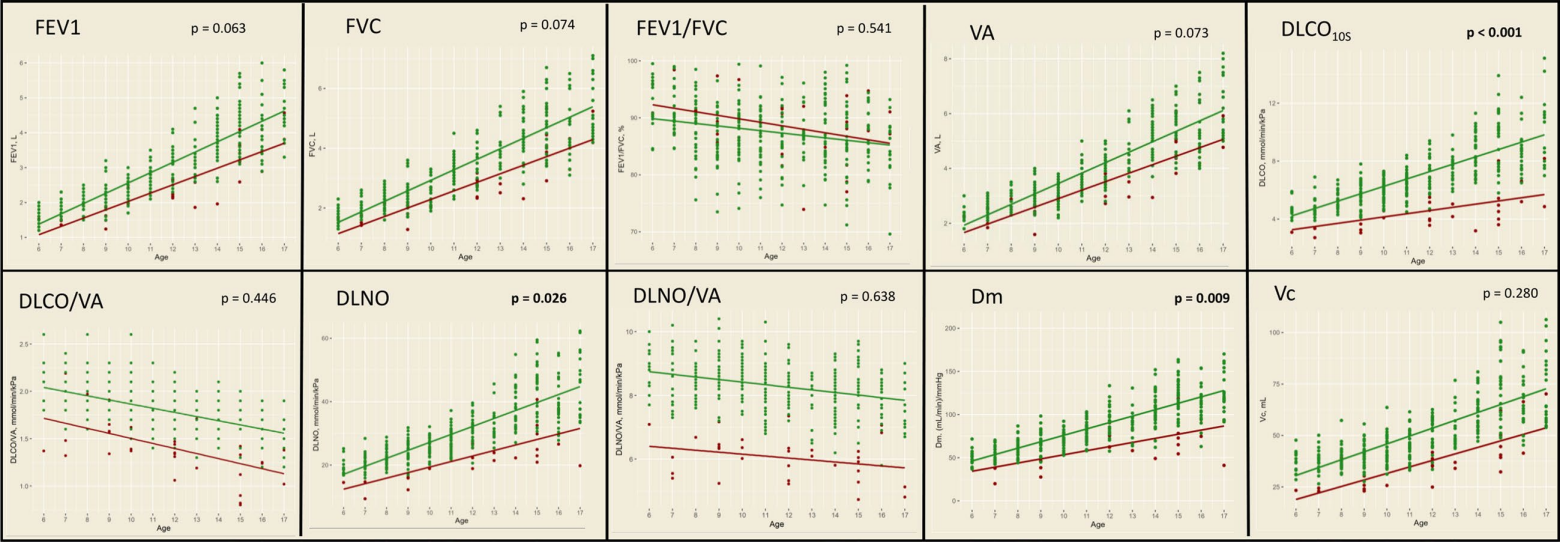
Comparison of cross-sectional PFT-I results in the pediatric Fontan group with reference data from 297 healthy Danish children [24]. Linear regression lines for the relationship between age and lung function measurement are shown, with red lines representing Fontan patients and green lines representing healthy children. The *p* value indicates the difference between the regression lines, calculated using interaction analyses with linear regression. *FEV1* forced expiratory volume in 1 s, *FVC* forced vital capacity, *VA* alveolar volume, *DLCO*_*10s*_ diffusing capacity for carbon, *DLCO/VA* coefficient of the lung for carbon monoxide, *DLNO* diffusing capacity for nitric oxide, *DLNO* transfer coefficient of the lung for nitric oxide, *Dm* membrane diffusing capacity, *Vc* capillary blood volume

Figure 1a presents the %pred results of spirometry and DLCO_10s_ in PFT-I and PFT-II in the pediatric group (*n* = 32), calculated with the GLI calculator. The median %pred spirometry measurements (FEV1, FVC, FEV1/FVC) all declined significantly, with the median %pred FEV1 declining the most, from 99.7 (IQR 92.4, 104.4) in PFT-I to 89.3 (84.9, 97.2) in PFT-II (*p* < 0.001). The median %pred VA also declined significantly, from 95.5 (IQR 89.5, 101.6) in PFT-I to 89.5 (IQR (79.7, 93.2) in PFT-II (*p* < 0.001). The median %pred DLCO_10s_ showed a tendency towards a decline, from 71.4 (IQR 62.1, 81.6) in PFT-I to 69.1 (52.8, 77.8) in PFT-II (*p* = 0.062), while the median %pred DLCO/VA remained unchanged. Spirometry and DLCO_10s_ results from the pediatric group are also found in Supplementary Table 1. Because of the lack of suitable reference equations for DLNO results in the pediatric group, the longitudinal data are not presented. Instead, cross-sectional data from PFT-I were compared with the Danish pediatric reference material. When comparing the results of PFT-I alongside PFT measurements from the Danish pediatric reference material, differences in the development of lung function in relation to age between the two groups became evident, see Fig. 2. Interaction analyses using linear regression found that DLCO_10s_, DLNO, and Dm increased significantly less with age in the pediatric Fontan group compared to healthy children. FEV1, FVC, and VA showed a similar trend, in spite of not being significant. The development of FEV1/FVC, DLCO/VA, and Vc showed no significant differences between the Fontan patients and the reference material.

### Pulmonary Function Tests in the Adult Group

**Fig. 3:**
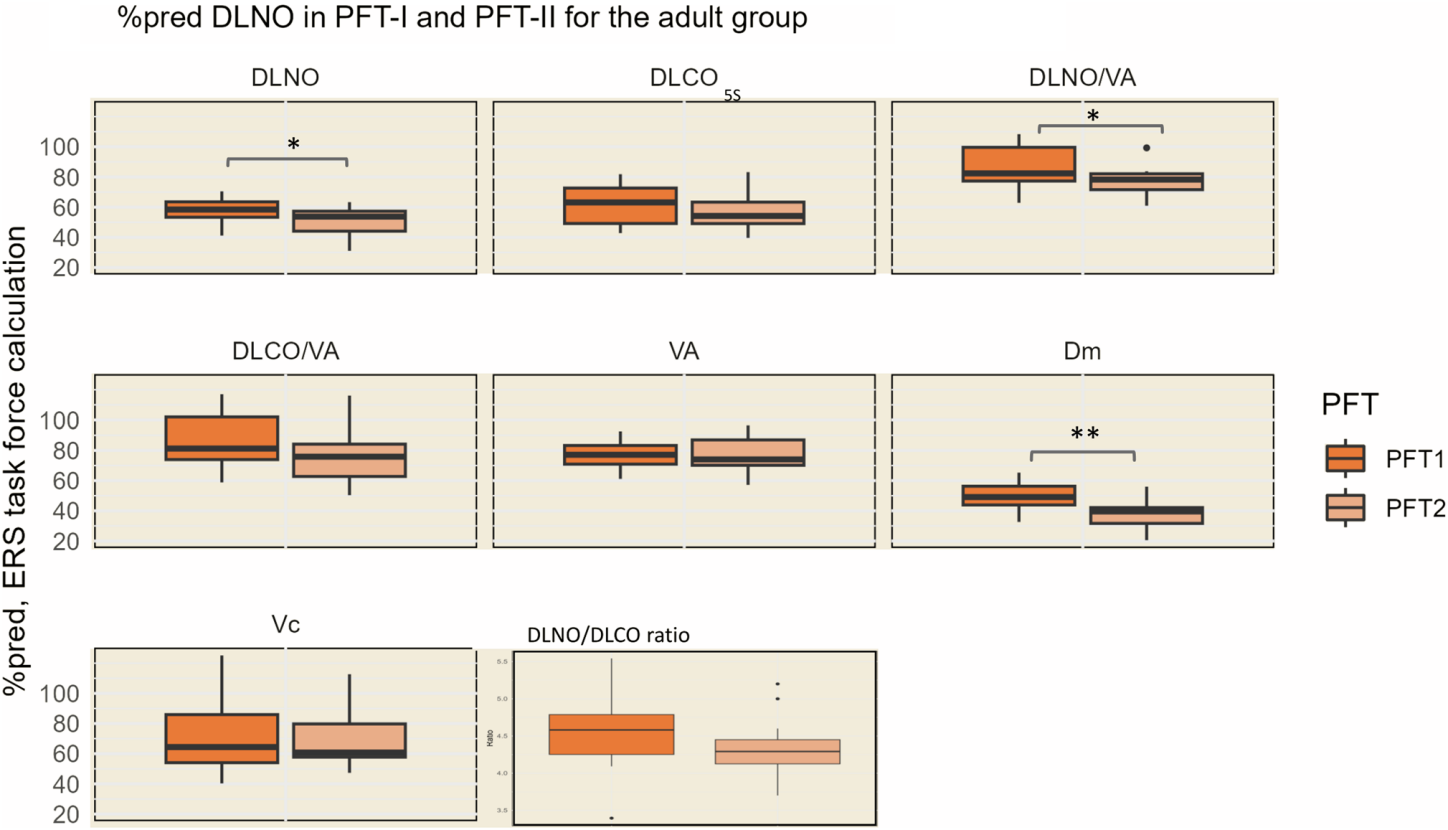
Results from the single-breath determination of nitric oxide uptake (DLNO) in PFT-I and PFT-II in the adult group are presented as percent predicted (%pred). The %pred values are calculated using the reference equations provided by Zavorsky et al. [20] in the ERS task force report. DLNO/DLCO ratio is reported as the ratio between the absolute measurements of DLNO and DLCO_5s_. Wilcoxon signed-rank test for paired data was conducted to check for significant differences from PFT-I to PFT-II (**p < 0.001, *p < 0.05). *DLNO* diffusing capacity for nitric oxide, *DLCO*_*5s*_ diffusing capacity for the lungs of carbon monoxide—5 s measurement, *DLNO/VA* transfer coefficient of the lung for nitric oxide, *DLCO/VA* DLCO corrected for alveolar volume, *VA* alveolar volume, *Dm* membrane diffusing capacity, *Vc* capillary blood volume

In the adult group (*n* = 16), there were no significant changes from PFT-I to PFT-II in any of the %pred results calculated with the GLI calculator, see Fig. 1b. The DLNO results from PFT-I and PFT-II in the adult group are presented in Fig. 3. DLNO results for the adult group are also shown in Supplementary Table 2. All %pred values are calculated using the reference equations by Zavorsky et al. [20]. The median %pred DLNO, DLNO/VA, and Dm all declined significantly during the study period, with the %pred median Dm declining the most from 49.1 (IQR 43.9, 56.3) in PFT-I to 39.6 (IQR 31.6, 42.1) in PFT-II (*p* < 0.001). In spite of not being significant, The DLCO_5s_ also tended to decline, median %pred 63.1 (IQR 49.2, 72.6) in PFT-I and 54.2 (IQR 49.1, 63.3) in PFT-II, *p* = 0.051. The development of the DLCO_5s_/VA and the DLNO/DLCO_5s_ ratio also showed tendencies towards a decline, although neither was significant. The median %pred VA and Vc remained stable during the study period. When looking closer at the DLNO components, the Dm was significantly more reduced than the Vc in both PFT-I (median %pred Dm 49.1 (IQR 43.9, 56.3) vs. median %pred Vc 64.4 (IQR 54.1, 85.9), *p* = 0.005) and in PFT-II (median %pred Dm 39.6 (IQR 31.6, 42.1) vs. median %pred Vc 60.7 (IQR 57.8, 79.8), *p* =  <0.001).

### Predictors of Lung Function

Simple regression analyses were conducted to identify associations between %pred PFT measurements at PFT-II and various clinical characteristics (see Supplementary Table 3). Among the 48 Fontan patients included in the regression analyses, 47 patients also performed a CPET at the time of PFT-II, why %pred VO2_peak_ was included in the models. The PFT variables investigated were: %pred FVC, %pred FEV1, %pred VA, %pred DLCO (%pred calculated with GLI calculator), and %pred DLNO (%pred calculated with reference equations by Zavorsky et al. [20]). The %pred FVC, FEV1, VA, and DLCO were not associated with any of the clinical variables investigated (sex, age, ventricular morphology, Fontan type, age at Fontan completion, %pred VO2_peak_). In the simple regression models, %pred DLNO was associated with age, Fontan type, and %pred VO2_peak_, while age at Fontan completion was not associated with %pred DLNO. In a multivariate regression model controlling for sex, age, and VA, a lateral or classic Fontan type remained negatively associated with the %pred DLNO, whereas a higher %pred VO2_peak_ was associated with a higher %pred DLNO (Table 3).

**Table 3:** Multivariate regression model investigating the relationship between percent predicted (%pred) DLNO in PFT-II and clinical characteristics

*n* = 48	DLNO %pred	
	Estimate ± SD	*p*
*Sex*	4.38 ± 2.18	0.051
Reference: Male		
Female		
*Age*	−0.36 ± 0.18	0.053
*%pred VA*	0.48 ± 0.11	**<0.001**
*Fontan type*	−7.93 ± 2.38	**0.002**
Reference: Extracardiac tunnel		
Lateral tunnel/classical Fontan		
*%pred VO2*_*peak*_	0.31 ± 0.11	**0.007**

Significance levels less than 0.05 are presented as bold

The %pred DLNO was calculated with the reference equation provided by Zavorsky et al. [20] in all patients

*DLNO* diffusion capacity for Nitric Oxide, *%pred* percent predicted, *VA* alveolar volume

## Discussion

In this longitudinal study with 10 years of follow-up on 48 Danish Fontan patients, we found significant declines in lung function over time. As hypothesized, the lung function decreased with increasing age but with different patterns in the pediatric and the adult group. The decline in %pred lung function was more pronounced in the pediatric group (under 18 years at PFT-I), with declines in both lung volumes and diffusion capacity—while only the diffusion capacity declined over time in the adult group (18 years or older at PFT-I).

### The Development of Lung Function in Fontan Patients

Studies investigating lung function in Fontan patients are limited, but it seems evident that impaired lung function is common [10, 11, 13, 14, 28]. In our study, we observed a restrictive lung pattern and a severely reduced DLCO and DLNO in both PFT-I and PFT-II, consistent with previous reports [29]. To our knowledge, no previous longitudinal studies are available for comparison of the development of lung function over time in Fontan patients. The most noticeable changes in lung function were seen in the pediatric group, with significant declines from PFT-I to PFT-II in the %pred FEV1, FVC, FEV1/FVC, and VA, in addition to a tendency towards a decline in %pred DLCO (*p* = 0.080). We interpret these declines in the %pred values as an impaired lung development during childhood and adolescence. It is well established that the lungs continue to develop from birth and into early adulthood [7]. Looking at reference data from healthy individuals spanning the transition from childhood to adulthood, a peak in lung function can typically be seen around 20 years [25, 27]. The lung function is highly dependent on height and, therefore, increases with age throughout the growth period. However, the growth spurt of the lungs has a lag compared to the growth spurt of the height, resulting in a continued increase in lung function into early adulthood [30]. As the pediatric group had a median age of 12 years at PFT-I and 23 years at PFT-II, we have captured many of these patients in a period with major growth, and hence, an increase in lung function. However, it may seem that lung function is not increasing as much as expected in our pediatric Fontan patients. A lower-than-normal increase in lung function in relation to age, would explain the declines in the %pred values in FEV1, FVC, FEV1/FVC, and VA in the pediatric group. The adult group, on the other hand, had a median age of 22 years at PFT-I. At this point, most had probably already completed their lung development, explaining why the corresponding %pred values remained stable in the adult group. Findings suggestive of abnormal lung function development in Fontan patients were also described in a 2018 study by Hedlund et al., where they observed a reduced increase in DLCO with age in 30 pediatric Fontan patients, compared with 25 healthy controls [28]. To further explore this theory, we compared the absolute measurements from PFT-I in the pediatric group with a reference material from healthy Danish children [19]. Figure 2 and its related interaction regression analyses support our theory: The difference in DLCO measurements between the Fontan patients and the healthy children significantly increased with age (*p* < 0.001), with a trend toward the same pattern for FEV1 (*p* = 0.063), FVC (*p* = 0.074), and VA (*p* = 0.073). This corresponds well with the %pred values that declined from PFT-I to PFT-II in the pediatric group. Although statistical significance was not reached in all the same parameters, this may be caused by the limited number of Fontan patients in the interaction regression analyses (*n* = 32).

### Reduced Lung Volumes in Fontan Patients

Several studies have found reduced lung volumes in Fontan patients [6, 11, 12, 28, 31]. Furthermore, reduced tidal volumes have been found in newborns with univentricular hearts prior to surgical interventions [32]. A post hoc sub-analysis including only patients under 10 years at PFT-I also found significant differences in FVC and VA when comparing Fontan patients to healthy Danish children. The decline in %pred FVC and VA from PFT-I to PFT-II results in further reductions in lung volumes compared to healthy peers as these patients reach adulthood. The impaired lung volume could, in part, elucidate the observed decline in diffusion capacity among Fontan patients, as a reduced lung volume translates to a diminished surface area available for effective diffusion. This is in line with novel findings in one of the few studies on DLCO in Fontan patients, in which Laohachai et al. concluded that the reduced DLCO in their patients was largely caused by a low VA [10].

### Reduced Diffusion Capacity in Fontan Patients

An impaired DLCO is found in various studies on Fontan patients, along with different theories on its causes [10, 11, 13, 28]. As mentioned by Laohachai et al. in their 2022 study, the assumption of a reduced VA as a cause of the reduced DLCO is opposed to findings in the first Danish study on our patient cohort by Idorn et al. from 2014, where the Vc seemed to be the primary cause of the reduced DLCO [14]. The only available reference equations at that time, however, were based upon older and relatively small reference materials [33–36], why we believe the new interpretation of the results from PFT-I gives a better understanding. When applying the newer reference equations by Zavorsky et al. [20] on our DLNO results, the Dm was significantly more reduced than the Vc in both PFT-I and in PFT-II—indicating that Dm also plays an important role. A more severe reduction in Dm compared to Vc indicates that the reduced diffusion capacity in Fontan patients likely is more affected by alterations to the alveolar membrane, than reduced capillary pulmonary flow [24]. Compared to healthy peers, both Dm and DLNO (partly a surrogate of Dm [20]) had a significantly lower-than-normal development during childhood and adolescence and an earlier-than-normal decline in adulthood. In contrast, Vc did not show age-related declines in neither the pediatric nor the adult group. Still, with a reduced Vc of about 70% of predicted in both PFT-I and PFT-II, as well as Idorn et al.’s findings of increased Vc and DLCO in the supine position [14], Vc likely also contributes to the reduced diffusion capacity in Fontan patients. Thus, it appears that the decreased diffusion capacity in Fontan patients is driven by impairments in VA, Vc, and Dm.

### The Pathophysiology of Impaired Lung Function in Fontan Patients

The exact pathophysiology behind the impaired lung function in Fontan patients remains unclear, but several concepts are discussed in a 2022 review by Laohachai and Ayers [29]. The negative impact of a non-pulsatile pulmonary flow on lung function is the most discussed theory. Matthews et al. proposed that a thickening of the alveolar capillary membrane caused by a non-pulsatile pulmonary flow could explain the reduced DLCO in Fontan patients in their 2006 study [11]. This assumption was partly made upon previous findings of medial hypertrophy in pulmonary arteries in Fontan lung biopsy specimens [37]. Since then, both abnormal vascular development [38] and endothelial dysfunction [39] have been associated with a non-pulsatile pulmonary flow. In a 2015 study by Ridderbos et al., adverse pulmonary vascular remodeling was found in lung tissue specimen from Fontan patients; decreased medial thickness and increased intimal thickness compared to control specimen were found in intra-acinar pulmonary vessels, with a significant correlation between the intimal thickness and the time since Fontan completion. The intimal thickening was mainly composed of acellular fibrosis and collagen deposition [38]. As intimal thickening also could be found with increasing age in the controls, but with a weaker relationship than in the Fontan patients, Ridderbos et al. proposed that the non-pulsatile flow in the Fontan circulation could accelerate and augment this aging process in the pulmonary circulation. These are interesting observations well-fitting with our findings of an earlier-than-normal decrease in the %pred Dm.

In the review by Laohachai and Ayers, it is further discussed whether an abnormal pulmonary flow can cause an impaired development of the alveoli as the patients grow [29]. Alterations in the pulmonary vasculature in Fontan patients begin in utero, as a result of the congenital heart malformation [5]. After birth, changes in pulmonary hemodynamics depend on the underlying anatomy and surgical procedures. The final result for all patients is the non-pulsatile pulmonary flow established at the Fontan completion, which often occurs around three years of age. During these first years of life, a massive development of lung parenchyma also takes place. The formation of the alveoli starts in utero, but the number of alveoli continues to increase at least during the first 2 years after birth [40]. Further growth of existing alveoli increases the alveolar volume up until early adulthood [4]. Given this development of lung parenchyma early in life, it seems plausible that the altered pulmonary hemodynamics found in Fontan patients could have a negative impact on alveolar volume. This is a noteworthy theory in relation to our findings. The VA was only slightly reduced in the pediatric group at PFT-I, at about 95% of predicted. However, the VA significantly declined in the pediatric group during the study period. The %pred VA did not decline in the adult group but was relatively stable, with a median %pred of 78% at PFT-II. Given these observations, we believe that the reduced VA found in the adult Fontan patients is primarily explained by impaired growth of the alveoli throughout childhood and adolescence—which could be secondary to unfavorable hemodynamics. Both the %pred Dm and DLNO did however decline in the adult group. It is, therefore, reason to believe that the hemodynamics of the Fontan circulation can negatively impact both the lung development during growth but also continue to negatively affect the alveolar capillary membrane after the lungs are fully developed.

### Predictors of Impaired Lung Function in Fontan Patients

In our regression analyses, DLNO was the only lung parameter found to be significantly associated with clinical parameters. As previously mentioned, the DLNO is mainly dependent of a well-functioning alveolar capillary membrane [20]. In our multivariate model adjusting for age, sex, and VA, %pred DLNO remained positively associated with %pred VO2_peak_, while a lateral tunnel or classic Fontan type was negatively associated with %pred DLNO. It is well established that DLNO is strongly correlated with VO2_peak_ in healthy people [41, 42], which we also found in our patients. Recent studies, including our own data [15, 43], suggest that increased physical activity can affect VO2_peak_ in Fontan patients. Physical activity might, therefore, have a more beneficial impact on lung function in Fontan patients than previously assumed. Previous studies have also reported associations between VO2_peak_ and FVC as well as FEV1 [6, 13], which we did not find, aligning with the more recent study by Laohachai et al. [10]. Laohachai et al., however, found an association between higher age at Fontan completion and abnormal lung volumes, which we did not find in this study. As the causes of lung impairment in Fontan patients seem to be multifactorial, and the patient populations often are heterogenous, this may explain the differing results.

## Conclusion

The impaired lung function in Fontan patients is multifactorial. We found declines in both lung volumes and diffusion capacity through the growth period in childhood and adolescence, indicating an abnormal lung development. Further, our findings of a decreasing diffusion capacity over time in both pediatric and adult patients, suggest that there is a continuous damage to the alveolar capillary membrane after creation of the Fontan circulation. Our findings emphasize further research on the lung function in Fontan patients, which should aim to elucidate the causes of impaired lung development, and the pathophysiology behind the decreased alveolar capillary membrane function.

## Limitations

Several limitations should be mentioned when interpreting our results. Firstly, our small cohort of only 48 patients with lacking longitudinal data on all 81 patients from PFT-I may not be representative of the Danish Fontan population. We further divided the cohort into a pediatric and an adult group to better interpret the results—which resulted in even smaller number of patients in some of the analyses. Efforts were made to present the results optimally by using the latest reference equations for %pred scores. Still, the calculation of %pred scores can bias the results if the reference equation is unsuitable for the population under study. Unfortunately, a suitable reference equation was not found for the %pred DLNO in those who transitioned from childhood in PFT-I to adulthood in PFT-II. Consequently, the longitudinal DLNO results were only presented in the adult group. This is a potential bias, as the DLNO analyses have a smaller sample size and higher average age than the full cohort of 48 patients. To describe the trends in DLNO results for the pediatric croup, we relied on cross-sectional reference data from healthy Danish children. However, these results are based on cross-sectional data and may be misinterpreted. Regarding the choice of a VIN/FVC cut-off at 80% for the DLCO, sub-analyses with the conventional cut-off at 85% were also performed. However, the main findings did not change—why the 80% threshold was kept, to include most patients in the analyses.

### Supplementary Information

Below is the link to the electronic supplementary material.

Supplementary file1 (PDF 158 KB)

## Data Availability

No datasets were generated or analysed during the current study.

## Abbreviations

DLCO: Diffusing capacity of the lungs for Carbon Monoxide
DLNO: Diffusing capacity of the lungs for Nitric Oxide
Dm: Diffusion capacity of the alveolar membrane
FEV1: Forced expiratory volume in 1 s
FEV1/FVC%: FEV1/FVC ratio
FVC: Forced expiratory volume
PFT: Pulmonary function test
VA: Alveolar volume
Vc: Pulmonary capillary blood volume
%pred: Percent predicted
