# Supplementary material for: Lung Function in Fontan Patients Over a Ten-Year Period: Is the Fontan Circulation Impairing Lung Development?
Source: Pediatr Cardiol. 2024 Jan 25;45(3):500–12. doi: 10.1007/s00246-023-03389-2 (PMC10891202; doi:10.1007/s00246-023-03389-2)
Supplement: Supplementary file 1 — Supplementary file1 (PDF 158 KB) [file 246_2023_3389_MOESM1_ESM.pdf]

Supplementary Table 1. Results from spirometry, lung volume, and $DLCO_{10S}$ in PFT-I and PFT-II, presented as median percent predicted (%pred). Patients are categorized into a pediatric (under 18 years at PFT-I) and an adult group (18 years or older at PFT-I). All %pred values are calculated with the GLI calculator. The Δ value indicates the change from PFT-I to PFT-II.

	**Pediatric group (n=32)**				**Adult group (n=16)**			
	**PFT-I** %pred	**PFT-II** %pred	**Δ**	**p**	**PFT-I** %pred	**PFT-II** %pred	**Δ**	**p**
**FEV1**	99.7 (92.4, 104.4)	89.3 (84.9,97.2)	-7.3 (-14.4,-2.7)	**<0.001**	84.2 (79.1,91.4)	87.1 (78.7,97.3)	0.8 (-8.1,6.8)	0.793
**FVC**	98.3(87.8,106.1)	96.7(86.7,100.6)	-5.8(-8.9,-0.3)	**0.008**	87.9 (82.7,92.7)	89.9 (83.3,100.2)	3.8 (-5.6,7.2)	0.298
**FEV1/FVC**	100.7 (96.7,105.2)	96.9 (93.8,100.7)	-4.8 (-6.7, -1.1)	**<0.001**	100.4 (93.8,104.6)	101 (93.1,102.3)	-2.3 (-5.5,0.3)	0.065
**$DLCO_{10S}$**	71.4 (62.1,81.6)	69.1 (52.8,77.8)	-5.2 (-13.6,4.6)	0.062	70.2 (52.4,74.5)	68.8 (56.7,76.3)	3.6 (-8.2,13.8)	0.562
**DLCO/VA**	74.5 (69.6,84.5)	81.9 (66.4,87.9)	2.1 (-5.9,14)	0.477	81 (62.6,89.8)	83.2 (75.1,92.7)	1.5 (-8.9,18.4)	0.274
**VA**	95.5 (89.5,101.6)	89.5 (79.7,93.2)	-8.7 (-16.3,-3.1)	**<0.001**	85.1 (78.7,90.1)	78.5 (74.6,92.6)	-3.9 (-9.2,0.2)	0.175

Data are presented as median (IQR)
FEV1 (forced expiratory volume in 1 second; FVC (forced vital capacity); $DLCO_{10s}$ (diffusing Capacity for Carbon Monoxide 10 seconds); DLCO/VA (DLCO corrected for alveolar volume); VA (alveolar volume)

Supplementary Table 2. Results from the single-breath determination of nitric oxide uptake (DLNO) in PFT-I and PFT-II in the adult group, presented as percent predicted (%pred). All %pred values were calculated using the reference equations published in the ERS task force report by Zavorsky et al. [21]

**Adult group n = 16**	**PFT-I**	**PFT-II**	**Δ**	**p**
**DLNO**	58.4 (53.3, 63.5)	53.7 (44.1, 57.3)	-7.7 (-10.6, 0.1)	**0.005**
**$DLCO_{5s}$**	63.1 (49.2, 72.6)	54.2 (49.1, 63.3)	-3.9 (-12.2, 0.9)	0.051
**DLNO/VA**	82.4 (77.3, 99.6)	78.3 (71.6,82.1)	-9 (-20.6, -2)	**0.006**
**DLCO/VA**	81.3 (73.9, 102.0)	75.9 (62.7, 84.2)	-10 (-24.2, 4.3)	0.058
**VA**	77.1 (70.9, 83.3)	74.0 (70.2, 87)	-2.1 (-6.3, 3.4)	0.597
**Dm**	49.1 (43.9, 56.3)	39.6 (31.6, 42.1)	-12.6 (-15.1, -7.7)	**p < 0.001**
**Vc**	64.4 (54.1, 85.9)	60.7 (57.8, 79.8)	3.6 (-5.5, 7.3)	0.706
**DLNO/DLCO ratio**	4.6 (4.3, 4.8)	4.3 (4.1, 4.4)	-0.3 (-0.5, 0)	0.083

Data are expressed as median (IQR).
DLNO (diffusing capacity for nitric oxide); DLCO5s (diffusing capacity for the lungs of carbon monoxide - 5 seconds measurement; KNO (transfer coefficient of the lung for nitric oxide); KCO (transfer coefficient of the lung for carbon monoxide); VA (alveolar volume); Dm (membrane diffusing capacity); Vc (capillary blood volume).

Supplementary Table 3. Simple regression models investigating the relationship between percent predicted (%pred) PFT measurements in PFT-II and clinical characteristics. The %pred values were calculated with the GLI calculator for FEV1, FVC, VA, and DLCO, and with the reference equation provided by Zavorsky et al. for DLNO.

	**FEV1** %pred		**FVC** %pred		**VA** %pred		**$DLCO_{10s}$** %pred		**DLNO** %pred	
**n = 48**	Estimate ± SD	**p**	Estimate ± SD	**p**	Estimate ± SD	**p**	Estimate ± SD	**p**	Estimate ± SD	**p**
**Sex**										
Reference: Male										
Female	6.17 ± 3.11	0.054	1.62 ± 3.40	0.636	0.45 ± 3.34	0.983	4.15 ± 4.15	0.322	4.73 ± 3.04	0.126
**Age**	0.03 ± 0.25	0.912	-0.05 ± 0.27	0.860	0.02 ± 0.26	0.948	0.43 ± 0.32	0.186	-0.50 ± 0.23	**0.035**
**Ventricular morphology**										
Reference: Left ventricle										
Right ventricle	5.78 ± 3.12	0.072	1.68 ± 3.40	0.624	-1.10 ± 3.35	0.744	-3.76 ± 4.16	0.370	-2.67 ± 3.09	0.392
**Age at Fontan completion**										
Reference: < 5 years										
> 5 years	4.88 ± 3.37	0.155	2.75 ± 3.60	0.450	-1.67 ± 3.56	0.641	7.43 ± 4.33	0.093	-1.07 ± 3.32	0.759
**Fontan type**										
Reference: Extracardiac tunnel										
Lateral tunnel/Classical Fontan	0.87 ± 3.45	0.802	-0.07 ± 3.62	0.985	4.48 ± 3.44	0.200	-2.32 ± 4.37	0.598	-7.61 ± 3.06	**0.016**
**%pred $VO2_{peak}$**	0.05 ± 0.16	0.747	0.13 ± 0.16	0.424	0.16 ± 0.16	0.320	0.20 ± 0.20	0.324	0.45 ± 0.13	**0.001**

FEV1 (forced expiratory volume in 1 second; %pred (percent predicted); FVC (forced vital capacity); VA (alveolar volume); $DLCO_{10s}$ (diffusing Capacity for Carbon Monoxide, 10 seconds measurement); DLNO (diffusing capacity for Nitric Oxide)
